# Building Respect, Responsibility, and Reciprocity: Insights From a Rural STEM Research Practice Design Partnership

**DOI:** 10.55533/2643-9662.1512

**Published:** 2025-07

**Authors:** Amanda Obery, Martha Cabell, Shelly Hogan, Matt Queen

**Affiliations:** Associate Professor of Elementary Math and Science Education at Central Washington University.; Evaluator with Yellowstone Evaluation Services.; Evaluator with Yellowstone Evaluation Services.; Associate Professor of Chemistry at Montana State University Billings.

## Abstract

Rural schools often have strong community involvement and thus can offer educational experiences to students that are more closely aligned with local people and places. However, curricula in rural schools rarely offer this same alignment. Partnerships with others, including higher education, can support rural schools in creating STEM curricula that center the resources and careers present in their communities. A research design practice partnership (RPDP) was created as a Third Space to enable the development of locally relevant STEM curricula. The partnership was formed focusing on respect, responsibility, and reciprocity, necessary tenets as the roles and goals of the project changed. Interviews with participants provide insights into the formation and maintenance of RPDPs, especially as roles changed and goals shifted. Collectively, the importance of having shared vision and goals for the project are a core part of establishing and maintaining respect. Responsibility is difficult to maintain within a Third Space, especially as members of long-term RPDPs change professionally. Reciprocity should be evidenced in many ways, starting at the onset of the RPDP, to establish a Third Space wherein all perspectives are valued and encouraged. Findings discuss the importance of, and challenges faced within, the RPDP, centering respect, responsibility, and reciprocity.

Rural areas often have rich educational systems that focus on local environments and resources and are supported with deep and wide-ranging community involvement (Avery, 2013; Lakin et al., 2021). Putting local people and places at the core of educational efforts, referred to as place-based education, helps students create a connection with their community (Sobel, 2004). Fostering family and community support in rural communities is key to developing positive attitudes, interest, and success for students, particularly in science (Cooper et al., 2005). Further, high-performing students in rural communities are often those with strong community attachment (Petrin et al., 2014, p. 320). It follows that a STEM curriculum explicitly centered on local knowledge (Avery, 2013) is likely to best support student success. Despite this connection, school curricula often do not consider rural perspectives (Biddle & Azano, 2016; Roberts, 2014, 2017).

Education in rural settings is not without its challenges, and many schools face issues regarding access to technology, teacher availability and training, and funding (Kormos & Wisdom, 2021; Nugent, 2017). The challenges can make it difficult for rural students and districts to receive support comparable to suburban/urban areas. To address some of these persistent issues, rural schools need external partners, such as higher education (Boyer, 2006). Research practice partnerships (e.g., Penuel, 2017) describe ways in which researchers and practitioners can work together toward a shared goal (Zeichner, 2010), leveraging the strengths of both partners. In this study, a research practice design partnership (RPDP) was formed as a Third Space to address long-standing challenges of making STEM curriculum of local relevance to rural communities (Figure 1).

Upper elementary teachers, district coordinators, and university faculty members collaborated for six years, at the time of this article, and their experiences speak to the dynamic power of rural RPDPs.

## Literature Review

### Rural STEM Education

Rural education is often viewed from a deficit perspective, one that ignores and neglects knowledge bases and rich contexts inherent in rural education (Avery, 2013). This deficit perspective includes mismatches between local values and career demands in that many specialized careers are not present in rural areas and parental expectations of educational attainment are lower (Harris & Hodges, 2018, pp. 3–9). However, family support and out-of-school engagement have been shown to be important factors in determining both STEM interest and persistence for students (Dierking et al., 2021; Harris & Hodges, 2018; Shaby et al., 2021). These factors stress the need to consider educational and career pathways in a holistic manner, one which considers the larger places and spaces that students occupy. Often referred to as “place-based education,” centering local people and places helps students connect to their communities (Sobel, 2004). A recent report from the National Academies of Sciences, Engineering, and Medicine (2024) emphasized that offering locally relevant initiatives that connect workforce and rural students’ everyday lives may lead to greater aspirations and interest in STEM (Starrett et al., 2022; Wingert et al., 2022).

Due to additional autonomy, rural teachers and administrators can be more flexible than their urban counterparts in their approach to supporting students’ STEM interests and aspirations (Lakin et al., 2021). This flexibility provides an opportunity to ensure local relevance in curriculum. As such, teachers and administrators are well suited to use the STEM ecosystems present in their communities to help initiatives in schools (Bhaduri et al., 2022). However, many rural teachers face challenges in teaching science, such as facilitating current science educational standards in a multigrade classroom (Hammack et al., 2022) and a lack of pedagogical or content knowledge (Zinger et al., 2020). Partnerships with local organizations and higher education may be beneficial for rural teachers and schools (Boyer, 2006) to address these challenges.

### Research Practice Partnerships

Creating spaces for change in education requires a multifaceted approach wherein invested people connect in meaningful and sustained ways to form a partnership. Using equity-based lenses (e.g., Penuel, 2017) to close the research-practice divide (Coburn et al., 2013; Farell et al. 2021), research-practice partnerships (RPPs) are one such approach to help address systemic challenges in education. Farrell et al. (2021) offered a definition of an RPP:

A long-term collaboration aimed at educational improvement or equitable transformation through engagement with research. These partnerships are intentionally organized to connect diverse forms of expertise and shift power relations in the research endeavor to ensure that all partners have a say in the joint work.

(p. 1)

Coburn et al. (2013) distinguished between three types of RPP: research alliances, design research, and networked improvement communities (p. 4). In the present study, an RPP was formed, focusing on the design of educational curriculum and materials, falling under the category of design research, so the partnership detailed herein is a research practice design partnership (RPDP).

RPDPs are often specific to one school district and aim to “develop materials and instructional approaches that can be implemented in classrooms, schools, and districts” (Coburn et al, 2013, p. 8). Researchers in RPDPs engage in work that improves the project as a whole, sometimes contributing to both theory and practice. To be successful, careful consideration must be made in defining the goals of the project and ensuring that problems of practice and revisions are an inherent aspect of the collaboration, with all parties invested in the work throughout planning and implementation (Coburn et al., 2013).

### Roles Within a Research Practice Partnership

Penuel et al. (2013) stressed the importance of defining roles within all RPPs to ensure a common focus. Making clear the ways in which practitioners and researchers function can help alleviate confusion and support the development of a successful RPP (Farrell et al., 2019).

Sjölund et al. (2022) conducted a review of roles within RPPs, using Coburn et al.’s (2013) three categories of RPPs as the structure for their analysis. About RPDPs specifically, Sjölund et al. (2022) described the work as collaborative and co-led by all parties, sharing stories of practitioners as designer pilots and validators and researchers as design leaders and advisors. “Practitioners and researchers work on more equal terms [within design partnerships compared to other RPP types] … as tasks are quite evenly distributed” (Sjölund et al., 2022, p. 1505).

Often the roles that researchers and practitioners take on within an RPDP challenge the norms of their respective spaces (Davidson & Penuel, 2019). Researchers, depending on their epistemological orientation, may maintain a distance between the research and the intervention. Teachers, depending on their experience, may not see themselves as research leaders. However, when committing to codesign processes within an RPDP, these traditional roles are no longer tenable. “Collaborative design, or ‘co-design,’ in design-based research is one strategy for leveraging the expertise of teachers to design, implement, and test educational innovations and, thereby, expand teachers’ agency within reform efforts” (Severance et al., 2016, p. 532), thus allowing for an RPDP to form. The roles assumed by partners change (Farrell et al., 2019), which is inherent in the process of collaboration and design within long-term sustained relationships, and role negotiation may occur frequently. In these dynamic spaces, trust between partners becomes centered (Henrick et al., 2017), and “when lines are blurred, a trust may develop that allows partners to see each other as critical to their own success” (Farrell et al., 2021, p. 23).

### Rural RPDPs as Third Spaces

RPDPs that form in rural spaces may offer a unique perspective on role negotiation, particularly if the researchers involved in the partnership come from outside the rural community. Considering learning through an ecological or ecosystems lens (Bhaduri et al., 2022; Bronfenbrenner, 1979), highlights the value of many rural educational systems in which communities are often deeply integrated into schools (e.g., Villa & Knutas, 2020). These tightly woven community connections can be the very reason researchers are drawn to rural places. However, earning the trust necessary to support shared knowledge is challenging for outside researchers as budgetary and time constraints may not enable the requisite space to create a meaningful bond (Anderson & Lonsdale, 2014, p. 195). As argued by Anderson and Lonsdale (2014), a focus on respect, responsibility, and reciprocity are important in the development of the research process, and the same principles can apply to RPDPs, though they challenge the dynamics established by Sjölund et al. (2022).

Drawing on the work of Bhabha (1994) as continued by Gutiérrez (2008), Third Space ideologies dismantle traditional hegemonies and normalized ways of being in favor of egalitarian systems. Collective Third Spaces are rooted in equity-based values as mediated by structures dictated by those who inhabit them (e.g., language and social dynamics; see Gutiérrez, 2008, pp. 148–151). Traditional approaches to research design practice partnerships have hierarchical structures, placing researchers as “design leaders” and community and teacher partners as “design pilots” (see Sjölund et al., 2022, p. 1498). Third Space recognizes the varied ways in which people view and make sense of the world, opening new opportunities, “where the potential for an expanded form of learning and the development of new knowledge are heightened” (Gutiérrez, 2008, p. 152). RPDPs often do not have the same work distribution as other RPPs (Sjölund et al., 2022) due to having a common goal in a problem of practice rather than research. Thus, RPDPs are likely more open to being hybrid or Third Spaces in which researchers and practitioners are not viewed as binaries (Daza et al., 2021; Zeicher, 2010).

To attain what Gutiérrez (2008) calls a “celebration of the local literacies” (p. 152), the activities leading toward the formation and maintenance of a rural RPDP as a Third Space necessitate Anderson and Lonsdale’s (2014) focus on respect, responsibility, and reciprocity. They conceptualize these three terms as:

1. Respect, wherein a two-way approach that values people, context, and place are established and maintained;
2. Cocreating responsible, honest, and transparent research and development processes;
3. Ensuring that the partnership is built upon a common reciprocal goal, having a strong connection to the people, context, and places it serves. (pp. 195–200)

Inherent in this vision is a requirement of the researchers to work “together” rather than “with” one another (Zeichner, 2010), blurring roles and creating spaces where knowledge from all participants is equally weighed and valued.

### Purpose

Rural teachers often face issues from funding to internet access (Kormos & Wisdom, 2021; Nugent, 2017), and RPPs can be an opportunity to collaborate with higher education and leverage strengths. An RPDP was formed as a Third Space to address challenges in the development of a STEM curriculum of local relevance to rural communities. Upper elementary teachers, district coordinators, and university faculty members worked collaboratively for years and continually navigated change. In this article, we explore the dynamic nature of the RPDP as a Third Space, created with respect, responsibility, and reciprocity.

## Method

This self-study navigated the complex interactions that occurred within a Third Space RPDP. The RPDP examined here includes teachers and researchers as co-designers and co-creators, Self-study, with its traditional practitioner-oriented lens (Williams et al., 2012), is justified and highlights the core role that teachers play in the RPDP.

### Context and Participants

Over the course of six years, the RPDP was formed in a rural state in the northern Rocky Mountains, between one administrator (curriculum director), nine teachers (grades 2–6) in the local school district, and two researchers (chemistry education and science teacher education) at a regional comprehensive university. The partnership began in 2019 with one administrator and the chemistry education researcher to better connect the schools and higher education, to cultivate interest in science, and to share local careers and places familiar to fifth-grade students. The goals of the partnership increased in scope and scale as opportunities emerged and a grant was secured to support the RPDP.

#### Timeline

Year 1: The district administrator for K–5 curriculum and the chemistry education researcher collaborated to link local schools and the university.

Year 2: The team expanded to include the science teacher educator. With a grant secured, a broader vision was set collaboratively with the district administrator.

Year 3: Six teachers joined the team (district administrator and researchers), creating an intentional RPDP. Biweekly meetings occurred for the first six months, trending toward a monthly meeting by the end of the academic year. Curriculum development and STEM educational materials were created and were piloted at the end of the year.

Year 4: Two teachers departed the RPDP, and three new teachers joined. STEM educational materials were edited, and advanced piloting took place in classrooms outside the RPDP.

Year 5: The district administrator moved to a different job, away from curriculum. Educational materials were revised again, and advanced piloting continued. Evaluators joined the team and began data collection.

Year 6: The team remained the same. The focus of the RPDP shifted to creating teacher training and supporting the full rollout of the curriculum.

The authors of this study are the researchers and evaluators of the overall grant, secured during year 2 of the project. The researcher-authors share a core belief in the value of STEM education and place-based pedagogies. Working with numerous rural communities, each of the researcher-authors have come to understand that rural communities, regardless of their economic basis, have a unique and enduring culture that uses STEM skills and knowledge differently (Moore & Yoho, 2023). Thus, the researcher-authors enter any new relationship with rural communities with humility and hope. In addition, the evaluator-authors included in this project deeply appreciate work in rural communities and have dedicated their organization to ensuring the integrity of projects in their state.

#### RPDP Structure and Products

The RPDP is part of a grant-funded project that allowed both the researchers and the teachers to be compensated for their time over the course of the project. Teachers were paid for three years and continue to receive payment for editing work and teacher training on an as-needed basis. Researchers continue to work with the administrator to support the full rollout of the curriculum to the district and lead teacher training. In addition, the grant project has two evaluators who are tasked with ensuring the project supports its intended mission. They also are involved in research that stems from the RPDP.

During years 3–5, when teachers, researchers, and a district administrator were compensated consistently for their involvement in the RPDP, the structure of the RPDP varied. During year 3, the RPDP met initially for two full-day sessions to brainstorm ideas, then met for a day and a half to establish working goals. Expectations for the RPDP (e.g., shared values, time commitments and consistent meetings, compensation, STEM fifth-grade curriculum aligned with the Next Generation Science Standards [NGSS]) were set during these primary meetings. The researchers only supported and asked probing questions when brainstorming ideas for the main focus of the STEM curriculum, but they took an active leadership role in discussing all other components of the project. The teachers and district administrator identified water as the most critical topic to the community. Thus, water and its filtration are the main STEM phenomena under focus in the curriculum and are used to meet the NGSS for fifth grade around matter and its interactions. A main activity (water filtration) and several anticipatory sets (e.g., videos of a person pretending to drink unfiltered water from the local river and water treatment facility interviews) were designed during these primary meetings.

The RPDP met on a biweekly basis for the first six months, with every other week being in-person and the other conducted via video conference. Explicit role negotiations occurred during the first few meetings, when no set leadership structure was in place. Several regularly scheduled meetings continued brainstorming without clear meeting facilitation or duties until one of the teachers stepped in and asked for more direction from the researchers. Researchers started to facilitate the meetings, create video content, and write lesson templates, while teachers decided to take ownership of writing individual lessons or lesson components. At the end of each biweekly meeting, the group would assign tasks to be completed in the interim.

Critical portions of the STEM curriculum, such as standards alignment and assessment work, were completed collaboratively during in-person meetings. The assessment meetings were run by one of the teachers who had experience with priority standards and district reporting, so the curriculum has a cohesive learning progression with aligned assessments. The draft STEM curriculum was completed in approximately six months and was piloted in the teachers’ classrooms. After each lesson was piloted, teachers completed an online review form, sharing tips and/or necessary edits to the lessons or components, which the researcher made.

In year 4, two teachers left the project (retirement and position change to an assistant principal), and three new fifth-grade teachers were added to the RPDP to fill the vacancies. This transition allowed for new perspectives, particularly around the clarity of lesson instruction. Meeting frequency for the RPDP decreased to monthly meetings to edit the drafted curriculum for an advanced pilot. Content background information was added to each of the lessons in the STEM curriculum as conversations in the RPDP focused on teachers’ need for support to teach the content. An advanced pilot was conducted, including classrooms outside the RPDP and without training, to test the feasibility and utility of the curriculum. As with the pilot, teachers were asked to provide feedback on implementation and suggest changes, after which the researcher made relevant edits.

In year 5, meetings were conducted on an as-needed basis, approximately bimonthly, to accommodate changes in administration and editing curriculum materials. The district administrator changed roles and no longer was responsible for elementary curriculum decisions, though they remained an advocate. In addition, they became a member of the grant advisory board, thus remaining active in the project if not a part of the RPDP. Also in year 5, two evaluators joined the grant project and supported the RPDP, conducting interviews with teachers to capture insights and reflections on the project’s progress. Now, in year 6, the STEM curriculum has moved into the district’s curriculum planning guide and is slated for full rollout due to the advocacy of the district administrator originally involved in the RPDP.

### Instrumentation/Measurement Procedures

Data were collected through semi-structured interviews with teachers and researchers involved in the curriculum development. All the district educators who participated in curriculum development were contacted, asked to interview, and offered compensation for their time. Seven of the nine current teachers and the two researchers were interviewed, for a total of nine interviews.

An interview protocol was developed with questions based on themes in the literature including the curriculum development process, challenges, roles, and perceptions related to RPDP. Interviews were conducted online by the two project evaluators to allow for easier transcription via an online video conferencing platform (either Zoom or Google Meet) and were audio recorded with participants’ consent. Interviews lasted 20–45 minutes. Platform-generated interview transcriptions were edited for accuracy.

### Data Analysis

The research team completed several rounds of coding using open coding and then thematic analysis (Savin-Baden & Major, 2013). The evaluators reviewed transcripts individually for initial open coding, after which the research team (two RPDP researchers and two project evaluators) reviewed codes to identify themes and, as a measure of trustworthiness and credibility (Savin-Baden & Major, 2013), had discussions to come to agreement. To enhance the transparency of the research, results were sent to all study participants to ensure their voices were accurately interpreted and reported. The evaluators who conducted the interviews also conducted these member checks to ensure that all participants could express their concerns openly and outside the RPDP.

## Results

Results from the nine interviews with RPDP participants are framed within Anderson and Lonsdale’s (2014) framework of emphasizing the importance of respect, responsibility, and reciprocity for working with rural settings. These constructs emerged prominently in interview responses and were necessary for establishing a Third Space within the RPDP. In addition to the thematic analysis of interview responses, the structural components of the rural RPDP are presented. These components are assessed based on their contribution to supporting or challenging the creation and maintenance of respect, responsibility, and reciprocity within the partnership.

### Respect

Respect, as an intersection of Zeichner (2010) and Anderson and Lonsdale’s (2014) work, centers on working together through a two-way approach in which all participants’ ways of knowing are included and privileged. In rural settings, this concept extends to include people and their broader contexts (Anderson & Lonsdale, 2014).

#### Values People

Establishing an RPDP on the grounds of respect was important to the researchers. One researcher said, when you are working in places that you are not familiar with, communities that you’re not familiar [with]…, researchers have the absolute risk of being irrelevant and invalid without [including] people who are on the ground, doing the day-to-day work, and being in touch with people there.

Another researcher stated that respect was important “Because teachers know everything.” They elaborated, saying, “[teachers’] depth of knowledge oftentimes draws on legacies within the community … and they see [students] lived realities … in the classroom. So they have a much more nuanced picture of what’s important [to students] than I do.”

For people in the RPDP, making these sentiments explicit and then reinforcing them with actions, such as ensuring all ideas were highlighted, was essential to fostering a culture of respect. The teachers were actively aware of these steps, with one saying, “as a team in person…, it was just a collaboration session, all share ideas, ask questions, [and make] suggestions, and I think having it on a consistent basis was what helps make it feel that way.” Another teacher commented,

The makeup of the team was always like an equal partnership. Not just with myself, but with those teachers who may not be science content experts, but they really are fifth grade teaching experts. And everybody was as equal at the table as everything was developed.

One of the researchers, who led initial interactions in the group, shared that throughout the design process an important step was to decenter themselves:

I just shut my mouth…. [I tried] to make sure that everyone got a chance to say something…, trying not to center myself too much. The less I say means the more that the group kind of takes over and therefore preferences some voices vs. the other.

#### Values Context

Context in a rural RPDP means creating a shared reality around the expectations for the project outcome—in this case, the STEM curriculum. Establishing a shared goal for the outcome, including feasibility, was essential. Strong internal cohesion around the goal was quickly established, as evidenced by every single interviewee’s acknowledgment of the worth of the new curriculum to engage students in science and to “have fun with it,” as one teacher put it. For this RPDP, this cohesion was likely due to the teacher selection process. The administrator in the RPDP led the selection process and explained, “[We] wanted ones that we knew would do quality work but also wouldn’t be afraid to say that we needed things.” Further, the teachers who stayed involved throughout the RPDP often did because, as one teacher said, “I love to do it [for the] kids. Science is fun, science is interesting.”

The role of what one teacher called a “naysayer” became very important for focusing on the context in which the design was occurring. The STEM curriculum was to be implemented in a district with high teacher turnover and with teachers who held emergency licenses or lacked experience. The RPDP teachers were often responsible for supporting these new teachers and understood the questions and management issues that could arise. Further, the school district has specific expectations for curriculum timing and assessment reporting. Therefore, for the STEM curriculum to be feasible, teachers needed to share district-specific information that the researchers did not understand. One teacher said,

I think that the biggest challenge kind of goes back to why it’s so important for the researcher [to have] boots on the ground. The biggest challenge is understanding [the teachers’] time constraints, right? I’m a teacher [and I know] what the district constrains. What they are allowed to do, and they aren’t allowed to do, [and what] they may be willing to do.

While it might have been a goal for the researchers to help with “building something that works for [the students, the researchers] have had no clue what it would look like in the classroom,” one teacher said. As both the researchers brought energy to the project, one teacher said they were “not sure who is having more fun—kids or [the researchers],” the teachers needed to be able to push back within the design process. Having a teacher say “no” was important to keep the curriculum feasible, as one teacher explained.

I was able to make sure that we kept bringing the curriculum up to the professors.… A lot of the things they kept bringing up and talking about we had to keep saying were still too high. Yeah, fifth grade isn’t able to understand what’s going on. So, I think we were definitely needed.

While teachers felt like they needed to push back, since respect was established, they also felt, “We could reach out by phone [and] continue open communication [with the researchers]. I think that was a safety net for our team that we appreciate.”

### Responsibility

Ensuring the responsible development of curriculum and/or research, and a transparent and open process (Anderson & Lonsdale, 2014), is a more challenging task in RPDPs, with their more equal distribution of tasks (Sjölund et al., 2022).

#### Initial Role Shifts

In the RPDP, participants had to shift roles, both at the start of and throughout the partnership. At the outset, teachers needed to see themselves as curriculum developers, which was a shift in perspective. One teacher explained,

I mean, we’re not curriculum developers, most of the curriculum you see taught in schools—that’s been written and developed by people who that’s their job. So just getting to a level of relevance of rigor tied to objectives and standards—I mean it’s a lot of work to do that.

Another teacher stated, “I thought we were going to just be doing some curriculum…, and then [the researcher] explained what the whole process was…. I had no idea [what] I signed up for.” Many of the teachers saw themselves as having a passive role in the process, rather than as contributors to the goal of cocreating “our own curriculum,” as one teacher said.

As the project continued, the researchers also had to reevaluate their roles. In response to a question about what they did or did not do to support a sense of ownership in the RPDP, one of the researchers, who came into the project with the idea that the teachers would run the group, said, “I … just didn’t pick up on it. My lack of facilitation from the start and putting in really clear expectations around roles [discouraged transparency].” The researcher shifted their role after one of the teachers spoke up and challenged the group dynamics, asking for direction.

#### Challenges With Role Adaptations

One area of challenge for the RPDP was being responsible, transparent, and open, as the RPDP adapted to teachers’ arrival and departure. As new teachers joined the RPDP, their roles were inherently different from those who started the work, and their inclusion shifted the nature of the group. One teacher who was added later said that their role was to refine what had been created, as opposed to feeling ownership of the curriculum. Another teacher, who joined in year two, felt they were included in “giving small opinions here and there.” In this long-term RPDP, where people shifted into and out of the partnership, roles and expectations were not navigated as well for participants who entered later as for those who started with the project.

#### Curriculum and Assessment

While the intent of the RPDP was to design curriculum, participants also created assessments for both district reports and research. Administrators brought in current content assessments, and researchers contributed reliable and valid affective and career measures of motivation. The administrator shared, “[the RPDP] got to write those assessment questions and adjust them. And again, it was just really fun to see that in year two [of the curriculum] adjusting for the needs of our district and our teachers.” Data from the pilots in years 3–5 were discussed at the meetings. One administrator said,

So I did look at some of those assessments. I think we’re getting closer to that content knowledge that we want. But really, for me, the greatest outcome is having kids start thinking about a future in science. Just that excitement about continuing on with science, thinking about potential careers in some of those fields to me, I think that’s more important than necessarily knowing the content.

As a result of data discussions within the RPDP, the assessments and research agenda changed, including adaptations to the assessment, the inclusion of interviews, and shifts in the timing of data collection not to coincide with springtime state testing.

#### Administrator’s Role

The responsibility of the administrator cannot be underestimated in a design-focused RPP. In year 5, the administrator involved in the RPDP changed roles. While remaining in administration, they are not the curriculum director. As a pivotal member of the RPDP, the administrator has supported the work from the beginning. Regarding this change, one of the researchers said, “I think actually the biggest change is going on right now. That has me the most nervous.” The researcher continued,

the difference between [the curriculum] just staying in that one classroom [vs. districtwide] … becomes, more often than not, administration [and] the degree to which they’re willing to support and push those [other teachers] who are very much … backing away. [The] more students … experience [the curriculum, the more] it becomes part of the culture.

The administrator remains an important advocate and shared, “we still have a pretty good solid plan” in reference to implementing the full curriculum to the remainder of the district. Their job transition has made full district implementation a longer process, as their time is directed in other administrative areas. However, because they share the core value in both STEM education and locally relevant curriculum, the administrator continues to advocate and support scheduling for implementation by working with the new curriculum director.

### Reciprocity

While Anderson and Lonsdale (2014) focused on reciprocity from a research perspective, within this RPDP, reciprocity can be seen through the project outcomes. Consistent reinforcement of the shared vision and careful consideration of the lasting outcomes in this study are at the core of reciprocity. Importantly, all curriculum from the RPDP remained with the district, including materials associated with the curriculum, and all participants were compensated for their efforts.

#### Community Relevance

Avery (2013) and Anderson and Lonsdale (2014) have pointed to the power of rural spaces and place-based educational systems, yet as one researcher noted, “rural communities … are oftentimes saddled with standardized curriculum that has very little to do with their ways of being.” Thus, crafting a locally relevant STEM curriculum that showcases people and places in their community was essential to the RPDP (Harris & Hodges, 2018; Sobel, 2004). Now, toward the end of the design process, a teacher shared, “I just love how we were able to tie in the community into the lessons, those video clips that make it so relevant, so I just think like that engagement for students. Community involvement engagement just making a lasting experience.”

When asked about the lasting impacts of the RPDP, one teacher said,

I think I’m hoping for two things. I think the lasting part of that is that understanding for the kids and the teachers how important the community is…, [and] I think empowering teachers to realize that they can create these community place-based education pieces and have the ability to do it…, Empowering the teachers to realize that they’re capable of it. It can be done. And kind of how we go about that process.

Beyond being capable of developing curriculum, the fact that the curriculum was created by teachers in the RPDP may ultimately be most important for its long-term implementation. A teacher reflected on this idea: “I also think it adds to the legitimacy of the curriculum, knowing that teachers within our own district had a hand in the development of it. And so teachers are more apt to listen to other teaching partners.”

#### Compensation and Appreciation

Compensation for efforts and outward displays of appreciation were given in reciprocity for all participants’ contributions to the project. Teachers were compensated for their time in set amounts for three years and as needed beyond that time. One participant shared, “[it] helped that there was a stipend that went with it…. On the days where we did meet, [a researcher] made sure teachers were treated really well [with] snacks, lunch.”

These findings illustrate how respect, responsibility, and reciprocity were foundational to the RPDP’s success and highlight the collaborative interactions among participants to create a Third Space.

## Conclusions

Core project outcomes are focused on harnessing the potential of place-based education (Sobel, 2004) to develop locally relevant STEM curriculum. Building on the resources and depth of knowledge present in the community (Avery, 2013; Lakin et al., 2021), the project aimed to foster interest in STEM and showcase local career pathways, an approach shown to be effective in rural areas (National Academies of Sciences, Engineering, and Medicine, 2024). These priorities originated from a partnership initiated by one administrator and the chemistry education researcher, evolving over time to encompass curriculum development goals. An RPDP was formed between local administrators and teachers in collaboration with two researchers from a nearby regional university to address the challenges of developing a locally relevant STEM curriculum,

With a specific focus on creating a Third Space (Gutiérrez, 2008), this RPDP was initiated and maintained through respect, responsibility, and reciprocity (Anderson & Lonsdale, 2014). Over the course of six years, the RPDP’s participants changed when teachers shifted responsibilities or roles, and new members were added in their place. These changes both challenged and reinforced the need to maintain a two-way approach that centered people and contexts, transparent development and assessment processes, and shared goals and strong connection to the community (Anderson & Lonsdale, 2014, pp. 195–200).

Findings reinforce the pivotal role of respect in the formation and maintenance of rural RPDPs. From the beginning, engagement in critical self-reflection around one’s own capacity and positioning led to a much greater understanding of the value of all participants. From the researcher perspective, eschewing traditional roles of leadership (Davidson & Penuel, 2019) enabled teachers to openly share their knowledge of the lived reality of students and their capabilities. Some educators needed to recognize their expertise and adopt a curriculum developer mindset. How the initial and ongoing meetings were structured around full participation and involvement in the design process may have signaled an intention to create a Third Space where every contribution is valued (Zeichner, 2010). Creating shared goals, while respecting the unique context of curriculum implementation, was essential to the RPDP’s success. The team was selected for their content and pedagogical knowledge, as well as their willingness to share their voice. Strong relationships were formed quickly as every participant shared a common goal around engaging students in STEM. Because of open communication, teachers were willing to push back to ensure that the context of the project was respected when the researchers misunderstood the realities of the teaching environment specific to the area.

The RPDP was challenged in the areas of responsibility and transparency. As noted previously, when creating a Third Space for a more egalitarian system in which traditional models are discarded and all ways of being are privileged (Gutiérrez, 2008), it is more difficult to establish roles. Some teachers entered the partnership thinking they would just develop curriculum, and a researcher entered believing the teachers would lead the group. Navigating these roles, particularly as new teachers entered and administrator positions changed, required negotiation (Farrell et al., 2019). Missteps occurred as teachers entered the partnership without a clear understanding of expectations and researchers did not facilitate in effective ways. New teachers also were not onboarded in the same manner as the initial team, leading them to feel as if they did not have as much agency in the project’s direction. In addition, while the plans for dissemination were solid during the first five years of the project, full district implementation of the curriculum may require administrative support from outside the RPDP.

Considering reciprocity, all participants in the RPDP were compensated for their involvement and continued participation. Meetings were conducted regularly as the scope of the project demanded, and deliberate efforts were made to share gratitude and respect for participants’ contributions. All participants shared that their efforts were supported. The goal of creating a locally relevant STEM curriculum was met as the curriculum created featured local phenomena (Sobel, 2004) and showcased careers in the community, and initial data points to its success (Obery & Queen, 2024). Their provides evidence of the collective abilities of RPDPs to create effective community-based curricula (Avery, 2013) and to empower participants to realize their own capabilities. Particularly given issues with rural teacher training, using locally relevant curriculum created by teachers may be an effective way to enhance STEM instruction.

### Limitations and Implications for Future Research

As place-based education focuses on the people and places specific to one area (Sobel, 2004), this RPDP and its outcomes are inherently of one place and people. This circumstance is both a strength and a limitation. This study’s rural context and limited participant numbers constrain the generalizability of its findings. Future studies may seek to see if similar RPDPs in places with strong community identity may come to a similar conclusion.

Challenges to Anderson and Lonsdale’s (2014) notions of responsibility were evidenced in this RPDP, especially during the formation of the team and when changes occurred. Further reflection on RPDP formation and role negotiation within Third Spaces, particularly at the start or during shifts in the partnership, could address issues and improve future RPDPs. One way to check on the ongoing health of the RPDP may be by including consistent check-ins, like those put forth by Henrick et al. (2023).

This RPDP is evidence of effective STEM curriculum development between administrators, teachers, and two researchers. The project was initially driven by the power of place-based education (e.g. Sobel, 2004) in rural areas (e.g., Avery, 2013) and their desire for curriculum materials that reflect their community. Creating opportunities for students to learn STEM through locally relevant phenomena not only encourages them to identify locally available careers (Lakin et al, 2021) but to also experience the places and spaces around them as worthy of study (Avery, 2013). Future research focusing on students who experience locally relevant curriculum may illuminate the connections between curriculum, place, and future outcomes. Exploring the value of including other stakeholders, such as industry partners, to enhance the local workforce aspects (Starrett et al., 2022), or differences if the researchers held other positions (e.g., STEM content professors), may uncover more about role negotiation and/or curriculum effectiveness.

## Figures and Tables

**Figure 1:**
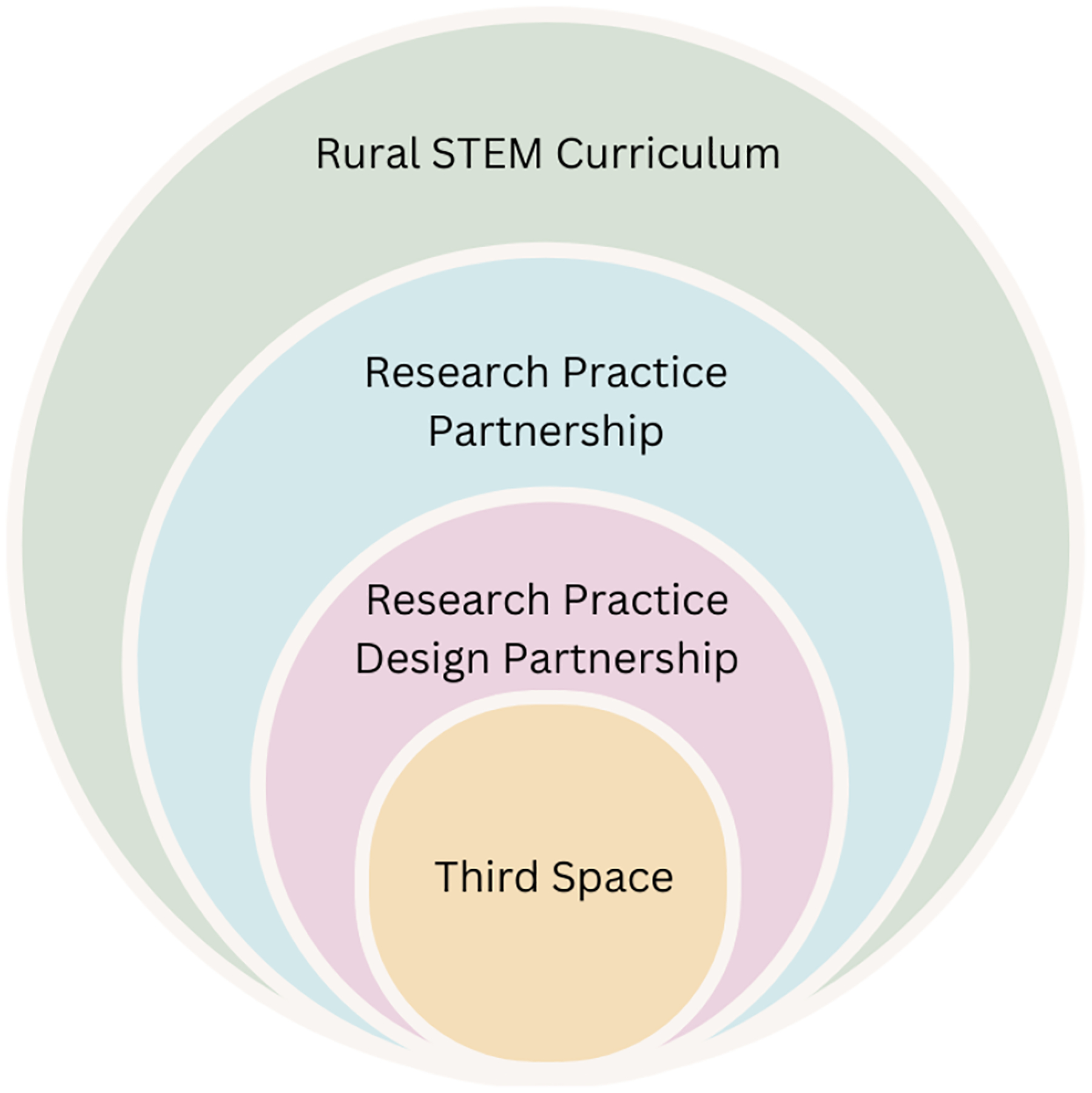
Study overview
